# Targeted Double Negative Properties in Silver/Silica Random Metamaterials by Precise Control of Microstructures

**DOI:** 10.34133/2019/1021368

**Published:** 2019-01-15

**Authors:** Peitao Xie, Zidong Zhang, Zhongyang Wang, Kai Sun, Runhua Fan

**Affiliations:** ^1^College of Ocean Science and Engineering, Shanghai Maritime University, Shanghai 201306, China; ^2^Key Laboratory for Liquid-Solid Structural Evolution and Processing of Materials (Ministry of Education), Shandong University, Jinan 250061, China

## Abstract

The mechanism of negative permittivity/permeability is still unclear in the random metamaterials, where the precise control of microstructure and electromagnetic properties is also a challenge due to its random characteristic. Here silver was introduced into porous SiO_2_ microsphere matrix by a self-assemble and template method to construct the random metamaterials. The distribution of silver was restricted among the interstices of SiO_2_ microspheres, which lead to the precise regulation of electrical percolation (from hoping to Drude-type conductivity) with increasing silver content. Negative permittivity came from the plasma-like behavior of silver network, and its value and frequency dispersion were further adjusted by Lorentz-type dielectric response. During this process, the frequency of epsilon-near-zero (ENZ) could be adjusted accordingly. Negative permeability was well explained by the magnetic response of eddy current in silver micronetwork. The calculation results indicated that negative permeability has a linear relation with* ω*^0.5^, showing a relaxation-type spectrum, different from the “magnetic plasma” of periodic metamaterials. Electromagnetic simulations demonstrated that negative permittivity materials and ENZ materials, with the advantage of enhanced absorption (40dB) and intelligent frequency selection even in a thin thickness (0.1 mm), could have potentials for electromagnetic attenuation and shielding. This work provides a clear physical image for the theoretical explanation of negative permittivity and negative permeability in random metamaterials, as well as a novel strategy to precisely control the microstructure of random metamaterials.

## 1. Introduction

Metamaterials, with unique negative electromagnetic parameters (e.g., negative refraction, permeability and permittivity), have gained extensive research attention due to their original applications in cloaking, waveguide, wireless power transfer, electromagnetic shielding and high-permittivity capacitor, etc. [1–5]. Metamaterials typically consist of artificially periodic array structure with different geometrical configuration (split-ring resonators, wires, fishnet, or cut-wire pairs) [1]. Rather than originating from the component materials, the unique property of metamaterials is fundamentally attributed to the resonance in these array structures [6, 7]. By designing the size and arrangement of the array units according to the applied wavelength, the electromagnetic resonance can be efficiently controlled to achieve the desirable electromagnetic properties of the metamaterials.

In fact, negative electromagnetic parameters have also been observed in some conventional materials, such as semiconductors and ferrite [6, 8]. By introducing these materials with optimal negative electromagnetic parameters into metamaterials according to the specific application requirement, the artificial difficulty in designing the geometrical configuration and arrangement mode of metamaterials as well as achieving the multifunctionalization of metamaterials can be obviously reduced for physicists and information scientists. Therefore, how to combine the conventional materials with metamaterials also becomes a hot issue in the field of materials science and engineering. At the same time, this can also expand the scope of metamaterials and develop the design methods of metamaterials. Zhou* et al.* pioneered this idea by introducing ferromagnetic materials and Mie resonance into metamaterials or photonic crystal to realize negative permeability or refraction [9–11]. Peng* et al.* observed the negative electromagnetic parameters in amorphous-metal sensors and transducers, a great success of multifunctionalization of the devices [12, 13].

Specifically, Fan* et al.* investigated negative permittivity and/or negative permeability in random percolative composites where conductive functional fillers were randomly distributed in the insulating matrix [14–16]. In these so-called intrinsic/random metamaterials, the sizes of the metamaterials are no longer dependent on wavelength, because the negative electromagnetic parameter directly originates from the intrinsic property of component materials. Besides, the random metamaterials, different from the periodic metamaterials, can be fabricated via the typical processing of materials where their properties can be efficiently controlled by changing the chemical compositions and microstructures of the component materials, which develops a novel and flexible way of adjusting negative electromagnetic parameters in the metamaterials [17–19]. As a result, random metamaterials have aroused tremendous interests in recent years [20, 21]. However, the distribution of functional fillers is usually random in percolative composites, a characteristic of percolation phenomenon [22]. The obtained electromagnetic properties, therefore, cannot be precisely controlled, which is not beneficial to the design and application of random metamaterials. Moreover, the mechanism of negative permittivity and permeability is still not clear enough in random metamaterials, as the random microstructure leads to the difficulty in studying their mechanisms. To address these issues, a novel strategy needs to be developed to precisely control the microstructure of random metamaterials, where the functional fillers can be accurately introduced into the targeted positions in the random metamaterials.

Herein we employed a self-assemble and template method to fabricate the Ag/SiO_2_ composite to achieve negative permittivity and permeability. Ag is a perfect candidate to provide the electromagnetic response, as it is an outstanding conductor due to the high electric conductivity (6.305×10^5^ S·cm^−1^). To provide the porous template, the insulating SiO_2_ microspheres are used where the silver is introduced in and its microstructure can be precisely controlled by a facile (<450°C) impregnation-calcination process. Negative permittivity is attributed to the low-frequency plasmonic state of the low-dimensional silver network and can be adjusted by Lorentz-type dielectric resonance. Negative permeability comes from magnetic behavior of induced current loops in the silver network. These random metamaterials are potential candidates to innovate some traditional electromagnetic industries. Electromagnetic shielding of ultra-thin Ag/SiO_2_ random metamaterials, herein, was investigated by electromagnetic simulation, showing the advantage of random metamaterials that its size is no longer restricted by applied wavelength. The shielding effectiveness of the Ag/SiO_2_ composite can be greatly enhanced to 40 dB with only 0.1 mm thickness near the epsilon-near-zero (ENZ) frequency range (520 MHz). Compared to the typical electromagnetic shielding material, a better electromagnetic interference (EMI) shielding performance can be expected when using the negative permittivity materials or ENZ materials for the EMI shielding applications.

## 2. Results and Discussion

**Figure 1:**
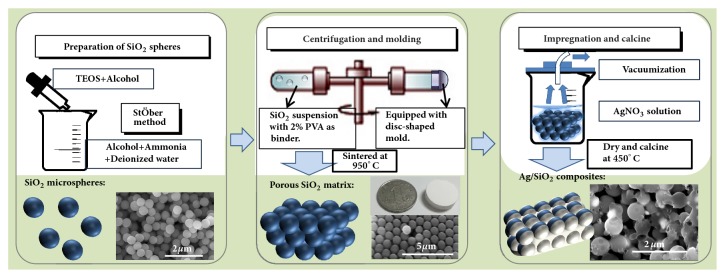
Schematic diagram of fabrication process of the Ag/SiO_2_ composites.

Figure 1 shows the main steps to fabricate the Ag/SiO_2_ composites. (1) The SiO_2_ microspheres were prepared by the typical Stöber method [23]. (2) The SiO_2_ microspheres were ultrasonicated in polyvinyl alcohol solution, followed by a centrifugation process to obtain a porous bulk sample. (3) The porous bulk sample was put into AgNO_3_ solution, which can fill in the interstices among the SiO_2_ microspheres. After that, the precursor composites were calcined to obtain the Ag/SiO_2_ composites.

### 2.1. Phase Characterization and Microstructure

**Figure 2:**
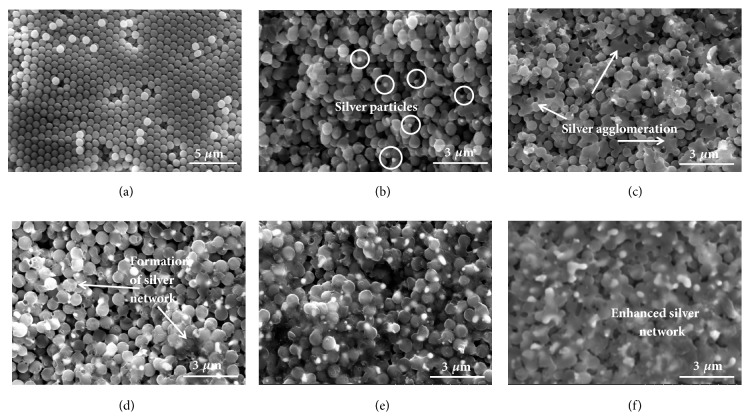
SEM image of SiO_2_ bulk (a), and Ag/SiO_2_ composites with 17 wt.% (b), 28 wt.% (c), 32 wt.% (d), 35 wt.% (e), and 37 wt.% (f) silver content.

The XRD patterns of the Ag/SiO_2_ composites illustrated the high purity of fabricated silver by the impregnation-calcination process (discussed in supporting information and Fig. [Supplementary-material supplementary-material-1]). The SEM images of Ag/SiO_2_ composites are shown in Figure 2. The diameter of SiO_2_ microsphere is about 700 nm, and SiO_2_ microspheres are closely arranged in the SiO_2_ bulk material (in Figure 2(a)). As we can see, the porous matrix can be constructed by the self-assembly method; the pore structure (size, shape, and connectivity) is no longer random but stable and repeatable. After the silver was introduced into the composites, the silver can only be distributed in the interstices among these SiO_2_ microspheres, where the silver nanoparticles will nucleate and grow during the calcination process. That is, the silver could be introduced into the targeted positions of the composites, which provides the feasibility to precisely control the microstructure of composites and adjust their electromagnetic property [19, 23]. When silver content is low, silver particles are isolated and randomly distributed in the composites with 17wt.% silver (Ag17) (in Figure 2(b)). With increasing silver content, the interconnection of silver particles is enhanced and silver agglomeration is formed (Figure 2(c)). Then three-dimensional silver network is formed in the channels among SiO_2_ microspheres in the composites with 32wt.% silver (Ag32) (Figure 2(d)). The network is gradually enhanced with increasing silver content (Figures 2(e)-2(f)). The conduction behavior is very sensitive to its microstructure according to percolation theory; hence, their conduction behaviors are investigated as follows [24].

### 2.2. Ac Conductivity and Percolation

**Figure 3:**
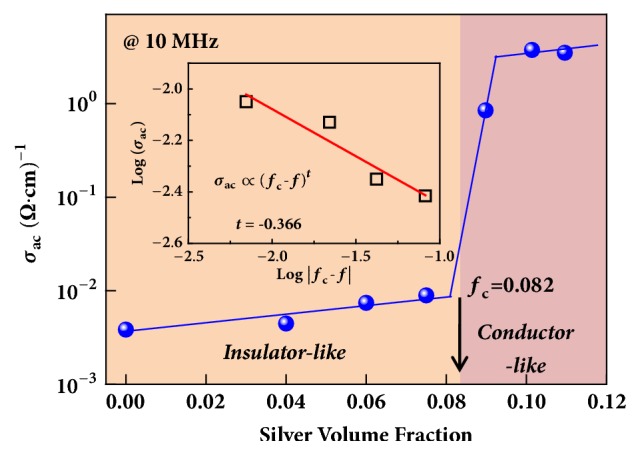
The variation of *σ*_ac_ (at 10 MHz) for Ag/SiO_2_ composites with silver volume fraction. The inset shows the calculation results using percolation theory: the percolation threshold is 0.082; the percolation critical exponent is* t*=0.366.

Figure 3 shows the variation of alternating current (ac) conductivity (*σ*_ac_) (at 10MHz) of Ag/SiO_2_ composites with different silver contents. The *σ*_ac_ gradually increases with increasing silver content when silver volume fraction* f* <0.08 but shows an abrupt increase near* f*=8 vol%. This is a typical percolation phenomenon, and the percolation threshold (*f*_c_) is near 8 vol% [14, 24]. The percolative behavior is attributed to the variation of microstructure as discusses above: when silver content is below *f*_c_, the silver particles are isolated by the SiO_2_ matrix and pore, while a continuous network could be formed when silver content is above *f*_c_. In this work, the percolative threshold was relatively low compared with the typical granular composites whose percolative threshold is usually 16 % [22]. In fact, the percolative threshold is influenced by the intrinsic property, shape, size, surface state, and dimension of the fillers. If the fillers are one-dimensional conductors, such as carbon nanotubes, carbon nanofibers, and silver nanowires, the percolative threshold can be decreased accordingly [22]. After the silver was distributed in the interstices among the SiO_2_ microspheres, the silver, to some extent, had a one-dimensional shape in the silver/SiO_2_ composites. Besides, the distribution of silver was restricted by the porous SiO_2_ microspheres template. The percolative threshold was, therefore, only about 8% in the silver/SiO_2_ composites. The abrupt change of conductivity can be expressed by classical percolation theory [22]:

(1) $$\begin{array}{c} \sigma_{ac}\propto {\left|\;f_{c}-f\;\right|}^{t} \end{array}$$

where* f* is the volume fraction of silver filler, *f*_c_ is the percolation threshold, and* t* is the corresponding critical exponent. The calculation results (inset of Figure 3) using Expression (1) show good agreement with experimental data, suggesting that percolation theory is applicable to the Ag/SiO_2_ composites. The frequency dispersion of *σ*_ac_ indicates that hopping conductivity behavior below the percolation threshold, while a metal-like conduction behavior is observed above the percolation threshold (detailed in the supporting information and Fig. [Supplementary-material supplementary-material-1]). Therefore, the change of electrical mechanism occurs in Ag/SiO_2_ composites, which can provide the feasibility of achieving the desirable negative permittivity property, studied in the following section.

### 2.3. Permittivity and Impedance

**Figure 4:**
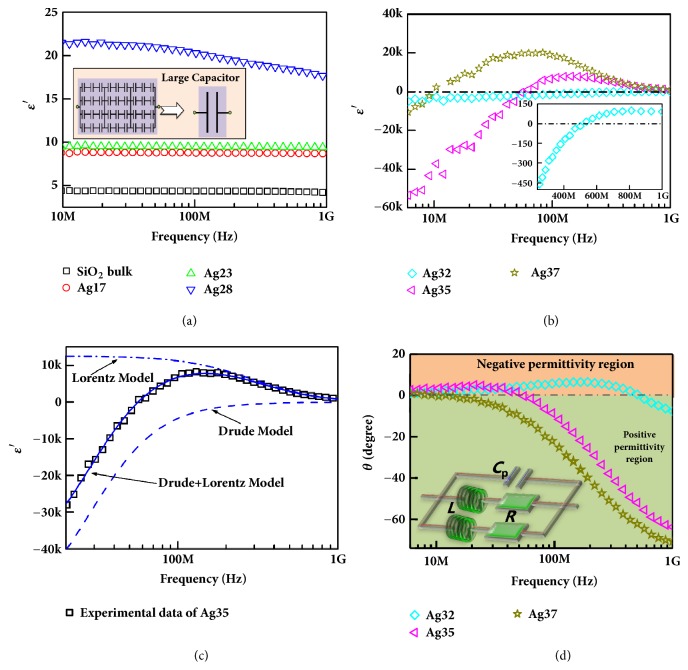
Frequency dispersion of real permittivity (*ε*′) for Ag/SiO_2_ composites (a, b); permittivity dispersion of Ag35 with calculation results (c). Frequency dependence of phase angle* θ *for Ag/SiO_2_ composites with different silver content (d). The dashed line is the calculated curve only by Drude model, the dash-dotted line is the calculated curve only by Lorentz model, and the solid line is calculated curve by both. The inset in (d) is the equivalent circuit of composites when the silver content is above percolation threshold.

Frequency dispersions of real permittivity (*ε*′) are shown in Figure 4. Permittivity shows obvious dependence on silver content. When silver content is below percolation threshold *f*_c_ (in Figure 4(a)), *ε*′ is positive and increases with increasing silver content, because the polarization across the silver/SiO_2_ interface indicates that any pairs of adjacent silver particles separated by an insulating gap can work as a microcapacitor [23]. The Ag/SiO_2_ composites can be regarded as a network of microcapacitors (inset of Figure 4(a)), which enables the composites to have a higher permittivity with increasing silver content. This behavior is also known as Maxwell-Wagner-Sillars effect [23], which is responsible for the enhancement of permittivity in heterogeneous composites. It is worth noting that the SiO_2_ bulk and composites with 17 wt.% and 23 wt.% silver content have potentials as novel high-permittivity materials over a broad frequency range, as their *ε*′ keeps steady in the whole test frequency region and their dielectric loss is relatively small with dielectric loss tangent tan⁡*δ* <0.01 (in Fig. [Supplementary-material supplementary-material-1]). When the silver content increases to 28 wt.%, the *ε*′ of the composites shows obvious frequency dispersion.

When silver content is above the percolation threshold *f*_c_, negative *ε*′ is achieved and shows obvious frequency dispersion (in Figure 4(b)). The *ε*′ is ~ -5000 at 6 MHz for composites with 32 wt.% silver content and gradually increases with increasing frequency. As for the composites with 35 wt.% silver content, the *ε*′ is ~ -50000 at 6 MHz and increases with increasing frequency, reaches the maximum (~8000) at 150 MHz, and then decreases with increasing frequency. As for the composites with 37 wt.% silver content, the *ε*′ is ~ -10000 at 6 MHz and keeps increasing to the maximum (~21000) at 68 MHz but then decreases with increasing frequency. The *ε*′ of Ag32 equals zero at 520 MHz, while at 57 MHz for the composite with 35wt.% silver (Ag35) and 11 MHz for the composite with 37wt.% silver (Ag37). That is, the epsilon-zero-point shifts to lower frequency with increasing silver content, which provides the feasibility of adjusting epsilon-near-zero properties. The Ag/SiO_2_ composites can not only be the promising candidate for negative permittivity but also be the epsilon-near-zero materials at radio-frequency region [25]. Generally, the plasma-type negative permittivity of metals can be expressed by the Drude model [17]:

(2) $$\begin{array}{c} \varepsilon^{\prime }=1-\frac{\omega_{p}^{2}}{\omega^{2}+{\Gamma_{D}}^{2}} \end{array}$$

where Γ_D_ is the damping constant, *ω*_p_=2*πf*_p_ is the plasmons angular frequency. Equation (2) is a monotonic increasing function, while the experimental data are not monotonic but have maximum (in Figure 4(b)). Because the experimental results cannot be perfectly explained by the Drude model only, there must be other mechanism contributing to the dielectric behavior of the composites [24]. The Drude model describes the simple harmonic motion of free electrons at altering electromagnetic field. Besides, the Drude model is usually used at the optical and infrared region to describe the negative permittivity. The frequency is very high; that is, period time is short, so the movement distance of the free electrons is also short. As a result, the plasma oscillation can be induced even in small metal particles. However, when the frequency of external electrical filed is low even in the radio-frequency region, free electrons will be localized in some small metal particles or agglomeration, leading to the deviation from the Drude model. The Drude model can describe the dielectric behavior of silver network only but is not applicable to isolated silver particles [21]. Since the isolated silver particles or agglomeration can lead to Lorentz-type dielectric behavior [24], the formula is modified after considering Lorentz-type dielectric resonance [24]:

(3) $$\begin{array}{c} \varepsilon^{\prime }=1-\frac{\omega_{p}^{2}}{\omega^{2}+\Gamma_{D}^{2}}+\frac{K\omega_{L}^{2}\left(\omega_{L}^{2}-\omega^{2}\right)}{{\left(\omega_{L}^{2}-\omega^{2}\right)}^{2}+\Gamma_{L}^{2}\omega^{2}} \end{array}$$

where Γ_L_ is the damping constant of Lorentz resonance, *ω*_L_=2*πf*_L_ is the Lorentz resonance angular frequency, and* K* is the dc electric susceptibility. As we can see from Figure 4(c) and [Supplementary-material supplementary-material-1], the calculation results (solid lines in Figure 4(c)) of Ag35 using Expression (3) show good agreement with experimental data with high reliability factor* R*^2^=0.99589. The Drude-type calculation results (dashed lines in Figure 4(c)) indicate that negative permittivity derives from the plasma-type dielectric behavior of free electrons in the silver network, while the Lorentz-type calculation results (dash-dotted lines in Figure 4(c)) indicate that the frequency dispersion of negative permittivity can be effectively adjusted by Lorentz-type dielectric resonance. These calculation results were made via the iterative method by using the respective model. Although Lorentz-type dielectric resonance can generate negative permittivity in some ferroelectric materials and composites containing carbon nanotubes [26, 27], the Lorentz-type dielectric behaviors have no contribution to the negative permittivity in Figure 4(b). In fact, it is a relaxation-type curve rather than resonance-type curve shown as dash-dotted lines in Figure 4(c), as the damping factor is much larger than resonance frequency (shown in [Supplementary-material supplementary-material-1]), a common phenomenon at low-frequency region in metal optics. The Lorentz-type dielectric behavior can be attributed to the interfacial imperfection (holes and voids) and the inhomogeneous silver distribution (isolated, agglomeration, and network) formed during adding silver into SiO_2_ matrix. It can come into the conclusion that the silver content plays an important role in controlling the frequency dispersion of negative permittivity.

Negative permittivity behavior is further investigated by phase angle (*θ*), as the positive permittivity is regarded as a capacitive character where the resistance current (*I*_R_) lags the capacitive current (*I*_C_) by 90°. When the silver content is above *f*_c_, the* θ* of Ag32 is positive below 520 MHz, below 55 MHz for Ag35, and 10.2 MHz for Ag37 (in Figure 4(d)), while the negative value of* θ* is above these frequency points. It shows that the inductive current (*I*_L_) lags the resistive current (*I*_R_) by 90°, which indicates that negative permittivity exhibits inductive character and the electric energy is stored in inductance [24]. Therefore, there were usually inductors in the equivalent circuit of negative permittivity materials because of the inductive character. The equivalent circuit analysis was performed by the ZSimpwin software. The optimal equivalent circuit was chosen with the smallest Chi-square value (usually 10^−3^-10^−5^). The Chi-square value represents the correlation between experimental data and simulated data. In the previous study, we have demonstrated that the distribution of conductive functional fillers can adjust the frequency dispersion of negative permittivity by LC resonance [23, 24]. In Figure 4(d), we can see that the silver content is crucial to the control of LC resonance because the value of* θ* is sensitive to the silver content. The absolute value of* θ* is very small in negative permittivity region, indicating that negative permittivity behavior has huge dielectric loss and potential application as loss materials. Besides, the absolute value of* θ* increases with increasing frequency in positive permittivity region, indicating the energy storage efficiency in capacitance is obviously higher than that in inductance [28]. Interestingly, the frequency points of* θ*=0 correspond with the epsilon-zero points. That is, there is no energy storage but energy loss at epsilon-zero point, indicating that Ag/SiO_2_ composites can function as resistor in metaelectronics circuits at radio-frequency region [29].

### 2.4. Permeability

**Figure 5:**
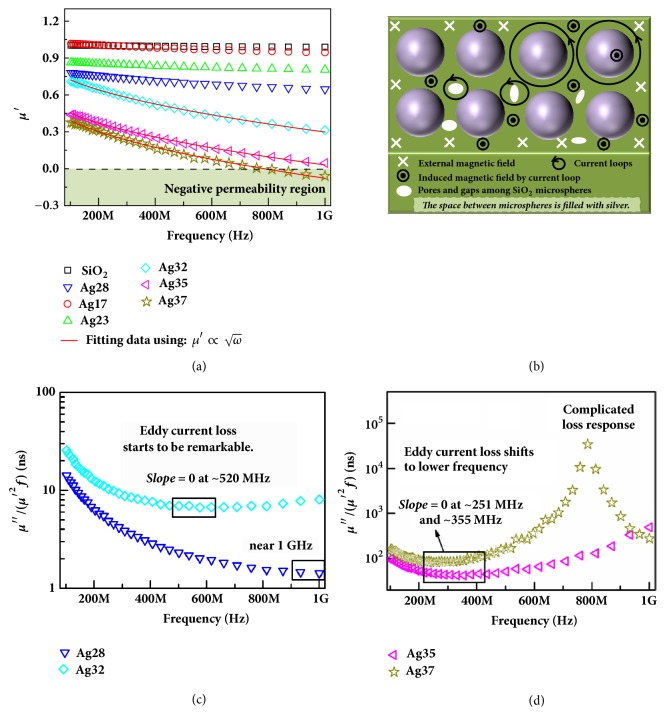
Frequency dependence of real permeability (*μ*′) for Ag/SiO_2_ composites (a), the schematic diagram of magnetic response due to the induced current loops in Ag/SiO_2_ composites (b), and frequency dependence of* μ*^″^/(*μ*′^2^*f*) for Ag/SO_2_ composites with different silver content (c, d). The red solid lines in (a) are the calculation results by the relation of $\mu^{\prime }\propto \sqrt{\omega }$ with high reliability.

Figure 5 depicts the frequency dependence of real permeability (*μ*′) property for Ag/SiO_2_ composites. As shown in Figure 5(a), the* μ*′ of SiO_2_ bulk almost keeps 1 due to its nonmagnetism. After silver is added to SiO_2_ matrix, the value of* μ*′ is smaller than 1, indicating negative susceptibility. The negative susceptibility is obviously enhanced with increasing silver content and frequency. When the silver content reaches 37 wt.%, negative permeability is achieved in the range of 760-1000 MHz. Since SiO_2_ and silver are both nonmagnetic, the negative susceptibility and negative permeability cannot be attributed to the magnetic resonance of component materials. It is worth noting that the previous investigations demonstrate that negative permeability is difficult to be realized in metal/ceramics random metamaterials that only contain nonmagnetic metal [17, 18]. Therefore, the generation of negative permeability indicates that the precise control of silver is in favor of the magnetic response of induced current, which improves the response efficiency and the possibility of realizing negative permeability. We can get some inspiration from periodic metamaterials as negative permeability is the unique property of periodic metamaterials, and its mechanism is derived from the law of electromagnetic induction [7, 30]. The split-ring resonators are the typical building blocks of magnetic response in periodic metamaterials, while the silver can work as building blocks of magnetic response in the Ag/SiO_2_ composites. As shown in the schematic diagram in Figure 5(b), the interstices among the SiO_2_ microspheres are filled with silver, and the current loops can be induced around the SiO_2_ microspheres when the composites are put under an alternating current magnetic field. The direction of the induced magnetic field from these current loops is opposite to the external magnetic field, which would cancel part of external magnetic field and thus leads to negative susceptibility [5]. When the induced magnetic field is even dominant, negative permeability will be achieved [7, 31]. Moreover, there are also many pores and gaps inside the silver network, where the microcurrents (small loops in Figure 5(b)) can also be induced to generate negative susceptibility/permeability.

According to the mechanism of negative permeability in typical periodic metamaterials, also known as “magnetic plasma”, the permeability of classical split-ring resonators (SRRs) can be expressed as follows [31]:

(4) $$\begin{array}{c} \mu_{eff}^{\prime }=1+\frac{F\omega^{2}\left(\omega_{0}^{2}-\omega^{2}\right)}{{\left(\omega_{0}^{2}-\omega^{2}\right)}^{2}+\Gamma^{2}\omega^{2}} \end{array}$$

where* F* is a geometrical factor,* ω*_0_ is the resonance frequency, and* Γ* is the resistive damping factor. The calculation data using magnetic plasma model are shown in Fig. [Supplementary-material supplementary-material-1] and [Supplementary-material supplementary-material-1]; the low reliability factors indicate that “magnetic plasma” cannot be the mechanism here. As shown in [Supplementary-material supplementary-material-1], the damping factors* Γ* are larger than resonance frequency by several orders of magnitudes, indicating a relaxation-type response in the view of “magnetic plasma” [14, 15, 18]. The relaxation-type behavior results in the huge deviation from “magnetic plasma”, primarily because of the great difference in shape and dimension between SRRs (typical “magnetic plasma” structure) and silver network (building blocks in this work). The capacitance from the split in SRRs leads to a resonance-type magnetic response, while no split structures are available in the silver network. In addition, it is well-known that the reported periodic metamaterials have* λ*/*l* value between 2 and 12, where* λ* is the wavelength and* l* is the size of the building block, while it is not applicable to this work [31]. Moreover, the relaxation-type response can be partly due to the small size of the silver network, since the damping factor is inversely proportional to the size of building blocks [32].

The “magnetic plasma” cannot explain the negative permeability/susceptibility, but the magnetic response can only result from the electromagnetic induction in the nonmagnetic composites. According to the Faraday's law, the induced current density can be expressed as follows [33]:

(5) $$\begin{array}{c} j_{i}=\sigma_{i}e_{i}=-\sigma_{i}\frac{d\Phi_{i}}{dt}=-\omega \sigma_{i}A\cos\left(\;\omega t\;\right) \end{array}$$

where *j*_*i*_ is the localized current density, *σ*_*i*_ is the ac conductivity of local area, *e*_*i*_ is the localized electric field intensity, Φ_*i*_ is the magnetic flux,* t* is the time, and* A* is the amplitude of *e*_*i*_. According to Biot-Savart law, the induced current loops can generate an induced magnetic field whose direction is always opposite to the external magnetic field. The magnetic response *B*_*i*_ at the axes of circulating current *j*_*i*_d*S*_*i*_ can be calculated by the formula [33]

(6) $$\begin{array}{c} B_{i}=\frac{\mu_{0}r^{2}j_{i}dS_{i}}{2\pi {\left(r^{2}+x^{2}\right)}^{3/2}} \end{array}$$

where *B*_*i*_ is the magnetic induction intensity,* r* is the radius of circulating current, d*S*_*i*_ is the sectional area of circulating current, and* x* is the distance to the center of circulating current. When the induced currents cannot keep up with the external field at very high frequency, the induced currents would lag and thus lead to an out-of-phase or negative response. The linear relationship between* μ*′, *B*_*i*_,* j,* and* ω* can be obtained: *μ*′ ∝ *B*_*i*_ ∝ *Nj* ∝ *ω*, where* N* is the winding number. Fig. [Supplementary-material supplementary-material-1] and [Supplementary-material supplementary-material-1] show the calculation results using linear relationship. The reliability factors (about 0.975) is fine but still cannot satisfy our expectation. Therefore, other factors should also be considered. In fact, the *σ*_*i*_ in Expression (5) should not be dc conductivity but ac conductivity. Ac conductivity is also the function of frequency, usually explained by skin depth *δ* = (2/*μ*_0_*σ*_*dc*_*ω*)^0.5^ [18]. The relationship between *B*_*i*_,* j,* and* ω* can be modified [16]:

(7) Bi∝Nj∝ω,μ′=a+bω

where* a* and* b* are parameters about the intrinsic property of Ag/SiO_2_ composites. The calculation results using (7) are shown as red solid lines in Figures 5(a) and [Supplementary-material supplementary-material-1], and the used parameters are in [Supplementary-material supplementary-material-1]. The calculation results agree well with experimental data (*R*^2^ ≈ 0.9975), indicating that the mechanism of negative susceptibility/permeability can be well explained [16]. The physical meaning of* a* is the initial permeability, and its value should be 1 for Ag/SiO_2_ composites, in good agreement with the calculation results (in [Supplementary-material supplementary-material-1]). The parameter* b* is an intrinsic parameter, which is closely related to the composition, microstructure and electromagnetic property of composites [34, 35].

The magnetic loss was also studied to reveal the influence of eddy current on negative susceptibility/permeability. The imaginary permeability (*μ*^″^) spectra for Ag/SiO_2_ composites are shown in detail in supporting information and Fig. [Supplementary-material supplementary-material-1]. Magnetic loss mainly originates from hysteresis loss, eddy current effect, and natural resonance at the radio-frequency region. The hysteresis loss is negligible in the weak field. Natural resonance can also be excluded due to nonmagnetism of Ag/SiO_2_ composites. Therefore, the eddy current loss is the only source of magnetic loss, and the eddy current loss can be expressed by [36]

(8) $$\begin{array}{c} \mu^{\prime \prime }=\frac{2\pi \mu_{0}\left({\mu^{\prime }}^{2}\right)\sigma_{dc}D^{2}f}{3} \end{array}$$

where* D* is the effective diameter of metal particles. If the magnetic loss simply derives from the eddy current loss, the value of* μ*^″^/(*μ*′^2^*f*) should be nearly constant without changing with frequency. Figures 5(c)-5(d) and [Supplementary-material supplementary-material-1] show the frequency dispersion of* μ*^″^/(*μ*′^2^*f*). When the silver content is below *f*_c_,* μ*^″^/(*μ*′^2^*f*) value obviously decreases with frequency (in Fig. [Supplementary-material supplementary-material-1]), there is an evidence of no eddy current loss available because the size of the silver particles is very tiny and even smaller than the skin depth. When the silver content is near *f*_c_ (i.e., Ag28 and Ag32), the value of* μ*^″^/(*μ*′^2^*f*), at the lower frequency region, also decreases with increasing frequency (in Figure 5(c)). Interestingly, the slope of Ag32 curve is zero at ~520MHz and at ~1GHz for Ag28, where* μ*^″^/(*μ*′^2^*f*) keeps constant, indicating that the eddy current loss gradually becomes dominant in magnetic loss [36]. Further increasing silver content, the slope of Ag35 curve is zero at ~355 MHz and at ~ 251 MHz for Ag37 (in Figure 5(d)), suggesting that the eddy current loss shifts to low-frequency region with the enhanced silver network. In fact, eddy current loss has a close relationship with the frequency of the external magnetic field, geometrical configuration, and the electrical conductivity of the metal. The faster the magnetic field changes, the stronger the eddy current loss can cause. In this work, the frequency shift of eddy current loss could not be attributed to the electrical conductivity of the metal, as the eddy current loss of all the composites came from the silver network. The only influence factor was the geometrical configuration of the silver network. From the SEM results (in Figure 2), it can be observed that the silver network was obviously enhanced with increasing silver content. That is, the thicker silver wires could be formed in the silver network. The electrical conductivity of the whole silver network could be enhanced with increasing silver content, and thus the remarkable eddy current loss could be caused at lower frequency region. It is worth noting that* μ*^″^/(*μ*′^2^*f*) increases with increasing frequency at higher frequency region (in Figure 5(d)). In addition, there is a loss peak for Ag37 curve at 760 MHz, and it also seems to be a peak for Ag35 at higher frequency region (above 1 GHz). These can be attributed to the complicated relaxation-type behavior from the phase lag of induced eddy current [31]. It is concluded that the eddy current effect is the main reason for negative susceptibility/permeability.

### 2.5. Electromagnetic Simulation and Shielding Effectiveness

Negative permittivity and permeability were simultaneously achieved in the Ag/SiO_2_ composites. In order to determine the interaction between electromagnetic wave and the materials, the electromagnetic propagation properties were simulated using Computer Simulation Technology software. The simulation method is detailed in supporting information and Fig. [Supplementary-material supplementary-material-1]. The shielding effectiveness (SE) is the main evaluation criterion of suppressing electromagnetic interference (EMI). The SE total (SE_T_) includes SE absorption (SE_A_) and SE reflection (SE_R_) [37].

**Figure 6:**
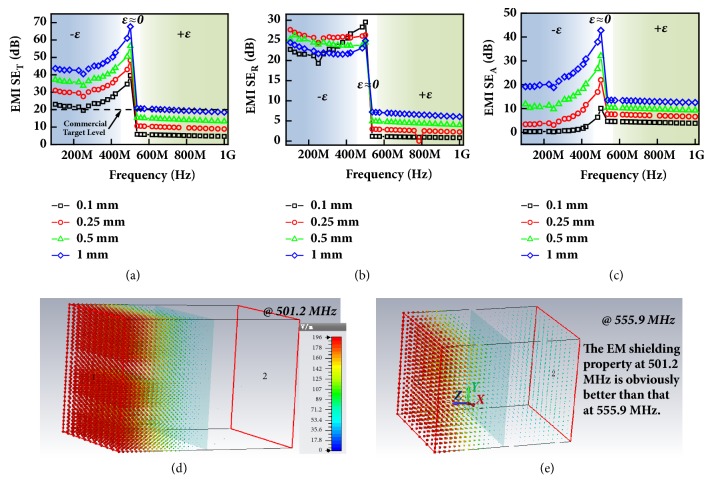
Frequency dispersion of EMI SE_T_ (a), SE_R_ (b), and SE_A_ (c) for Ag32 metacomposites with different thickness. The distribution of electric field vector of Ag32 composites at 501.2 MHz (d) and 555.9 MHz (e) with 0.25 mm thickness.

The simulated SE results of Ag32 were shown in Figure 6. The SE_T_ value increased with increasing frequency at 100-520 MHz, then the SE_T_ value dramatically dropped and kept a small value at 520-1000 MHz. Intriguingly, the frequency region with large SE_T_ value corresponds well with that of negative permittivity, while low SE_T_ corresponds well with positive permittivity region (in Figure 4(b)). Similar results were also observed for Ag35 and Ag37 in Fig. [Supplementary-material supplementary-material-1]-[Supplementary-material supplementary-material-1], which is because the formation of the silver network could lead to high SE value and excellent electromagnetic shielding property [38]. The SE_R_ value almost kept constant (22.5-27.5) at 100-520 MHz (in Figure 6(b)) and is independent on the thickness of composites, because the thickness of silver network is thicker than the skin depth of metal silver (0.064 mm at 100 MHz). In contrast, the SE_A_ value obviously increased with higher frequency and thicker slab at 100-520 MHz (in Figure 6(c)), a peak value near 520 MHz. The enhanced SE_A_ value had a close relationship with the epsilon-near-zero property of Ag32 composites, because of good impedance matching and absorption loss. Similar enhanced absorption phenomenon was also observed in indium tin oxide at infrared band [39]. Therefore, the epsilon-near-zero property is like an “on-off switch” of electromagnetic shielding, providing the possibility of intelligent frequency selection. It is further demonstrated by the distribution of the electric field vector in Figures 6(d)-6(e), where the electromagnetic shielding at 501.2 MHz is obviously better than that at 555.9 MHz. It is worth noting that the SE_T_ value of Ag32 can reach the target level of 20 dB for commercial application at 100-520 MHz, and the thickness can be as thin as 0.1 mm. We can conclude that excellent shielding effectiveness, with the advantage of enhanced absorption, intelligent frequency selection, and thin layer, makes the Ag/SiO_2_ random metamaterials highly competitive to conventional metals and carbon shielding materials [38, 40].

## 3. Discussion

Porous SiO_2_ template was prepared via self-assemble process. Silver was precisely introduced into the interstices among SiO_2_ microspheres to construct Ag/SiO_2_ random metamaterials by the impregnation-calcination process, functioning as functional fillers. The electrical conductivity results revealed that a percolation phenomenon occurred. The conductive mechanism changed from hopping conduction to Drude-type conduction, owing to the formation of silver networks with increasing silver content. Negative permittivity can be well explained by plasma-like behavior of the silver network and Lorentz-type behavior of silver agglomeration. Negative permeability was attributed to the magnetic behavior of induced current loops. The calculation analysis indicates that permeability values have a linear relation with* ω*^0.5^, which is different from the “magnetic plasma” of periodic metamaterials. Electromagnetic simulations demonstrated that negative permittivity materials and epsilon-near-zero materials can have potentials for electromagnetic attenuation and shielding.

## 4. Materials and Methods

### 4.1. Materials

The ammonium hydroxide (28 wt. %), ethanol, tetraethyl orthosilicate (TEOS), polyvinyl alcohol (PVA), and silver nitrate (AgNO_3_) were all purchased from the Sinopharm Chemical Reagent Co. Ltd. China. The chemicals are obtained as chemically pure grade products and used without any further treatment.

### 4.2. The Preparation of SiO_2_ Microspheres

The SiO_2_ microspheres were prepared by the Stöber method. 25 mL deionized water, 80 mL ethanol, and 20 mL ammonium hydroxide were mixed as A solution at 40°C. 40 mL TEOS and 80 mL ethanol were mixed as B solution. B solution was added to the A solution at a speed of 200 mL/h. Subsequently, the mixture was kept stirring for 12 h at 40°C. The SiO2 microspheres were obtained by centrifuging the white precipitation and further cleaned by deionized water for several times until pH=7.

### 4.3. The Preparation of Ag/SiO_2_ Metacomposites

3g SiO_2_ microspheres were dispersed in 50 mL 2 wt.% PVA solution. The SiO_2_ disks were prepared by centrifugal sedimentation method using the SiO_2_ suspension. The SiO_2_ disks can be obtained in the specially designed mold which placed at the bottom of the centrifuge tube and then dried at 60°C for 24 h. The obtained SiO_2_ disks were sintered at 950°C for 30 min to prepare porous SiO_2_ matrix. The porosity of SiO_2_ matrix was 32%. Porous SiO_2_ discs were soaked into the AgNO_3_ solutions of 2mol/L and vacuumed for 10 min to “press” the solution into porous SiO_2_. The discs were dried at 70°C for 3h then 100°C for 8 h. Finally, the discs were calcined at 450°C for 30 min to obtain Ag/SiO_2_ composites. The silver mass fraction was controlled by impregnation-calcination cycles.

### 4.4. Characterization

The XRD patterns were carried out by the Rigaku D/Max-RB-type X-ray diffraction target Cu (K*α* diffraction line). The morphologies of the fracture surface morphologies were obtained by scanning electron microscope (SU-70 Field Emission Scanning Electron Microscope, FESEM) equipped with energy dispersive X-ray spectroscopy (EDX). Dielectric parameters of the composites were measured by Agilent E4991A Precision Impedance Analyzer with 16453A dielectric test fixture from 1 MHz to 1 GHz, including impedance (*Z*′,* Z*^″^), capacitance (*C*), and resistance (*R*_p_), ac conductivity (*σ*_ac_), the phase shift angle (*θ*), and the real (*ε*′) and imaginary (*ε*^″^) part of complex permittivity. The test fixture of 16454A was used to measure the complex permeability. The dimension of the samples for permittivity measurement is *ϕ* 20 mm × 2 mm. The thickness was controlled by polishing the samples on abrasive papers (1200 mesh). The toroidal samples were used for permeability measurement with the dimensions of 6.5 ×20 × 2 mm. In order to prepare the toroidal samples, a small pore was made at the center of the samples by a drill (the diameter 1 mm), and then the small pore was gradually enlarged by a conical file.

## Data Availability

All data needed to evaluate the conclusions in the paper are present in the paper and/or the Supplementary Materials. Additional data related to this paper may be requested from the authors.

## Supplementary Materials

Supplementary Materials
** Fig. S1:** XRD patterns (a) of Ag/SiO_2_ composites with different silver content and EDX analysis results (b) of sample Ag23.** Fig. S2:** EDX analysis results of sample Ag23.** Fig. S3:** the frequency dispersion of *σ*_ac_ in 6 MHz–1 GHz region. The solid lines are calculation results using the power law and Drude model.** Fig. S4:** frequency dependence of imaginary permittivity* ε*′′ (a) and dielectric loss tangent tan⁡*δ* (b) of Ag/SiO_2_ composites with different silver content.** Fig. S5:** frequency dependence of phase angle* θ *for Ag/SiO_2_ composites with different silver content at 6 MHz–1 GHz.** Fig. S6:** Nyquist plots for samples SiO_2_ bulk, Ag17, and Ag23; their results of equivalent circuit analysis. The inset is the equivalent circuit for Ag/SiO_2_ composites with different silver content. The solid lines are calculation results using equivalent circuit.** Fig. S7:** the fitting results of permeability spectra of Ag32, Ag35, and Ag37 using magnetic plasma. The red solid lines are fitting data.** Fig. S8:** the fitting results of permeability spectra of Ag32, Ag35, and Ag37 by linear fitting. The solid lines are fitting data.** Fig. S9:** the calculation results of permeability spectra of Ag17, Ag23, and Ag28 by (7). The solid lines are calculation data with high reliability.** Fig. S10:** frequency dependence of imaginary permeability (*μ*^″^) (a) and *μ*^″^/(*μ*′^2^*f*) (b) for Ag/SO_2_ composites with different silver content.** Fig. S11:** the square slab model built for numerical simulation.** Fig. S12: **frequency dispersion of EMI SE_T_ (a), SE_R_ (b), and SE_A_ (c) for Ag35 composites with different thickness.** Fig. S13: **frequency dispersion of EMI SET (a), SER (b), and SEA (c) for Ag37 composites with different thickness.** Table S1: **the fitting results of ac conductivity using power law.** Table S2: **the fitting results of ac conductivity using Drude model.** Table S3: **the calculation results of real permittivity using Lorentz model.** Table S4: **the calculation results of equivalent circuit.** Table S5:** fitting results using (4).** Table S6:** fitting parameters of linear fitting.** Table S7: **the calculation results of (7).
