# Supplementary material for: Targeted Double Negative Properties in Silver/Silica Random Metamaterials by Precise Control of Microstructures
Source: Research (Wash D C). 2019 Jan 15;2019:1021368. doi: 10.34133/2019/1021368 (PMC6750100; doi:10.34133/2019/1021368)
Supplement: Supplementary Materials — Fig. S1: XRD patterns (a) of Ag/SiO2 composites with different silver content and EDX analysis results (b) of sample Ag23. Fig. S2: EDX analysis results of sample Ag23. Fig. S3: the frequency dispersion of σac in 6 MHz–1 GHz region. The solid lines are calculation results using the power law and Drude model. Fig. S4: frequency dependence of imaginary permittivity ε′′ (a) and dielectric loss tangent tan⁡δ (b) of Ag/SiO2 composites with different silver content. Fig. S5: frequency dependence of phase angle θ for Ag/SiO2 composites with different silver content at 6 MHz–1 GHz. Fig. S6: Nyquist plots for samples SiO2 bulk, Ag17, and Ag23; their results of equivalent circuit analysis. The inset is the equivalent circuit for Ag/SiO2 composites with different silver content. The solid lines are calculation results using equivalent circuit. Fig. S7: the fitting results of permeability spectra of Ag32, Ag35, and Ag37 using magnetic plasma. The red solid lines are fitting data. Fig. S8: the fitting results of permeability spectra of Ag32, Ag35, and Ag37 by linear fitting. The solid lines are fitting data. Fig. S9: the calculation results of permeability spectra of Ag17, Ag23, and Ag28 by (7). The solid lines are calculation data with high reliability. Fig. S10: frequency dependence of imaginary permeability (μ″) (a) and μ″/(μ′2f) (b) for Ag/SO2 composites with different silver content. Fig. S11: the square slab model built for numerical simulation. Fig. S12: frequency dispersion of EMI SET (a), SER (b), and SEA (c) for Ag35 composites with different thickness. Fig. S13: frequency dispersion of EMI SET (a), SER (b), and SEA (c) for Ag37 composites with different thickness. Table S1: the fitting results of ac conductivity using power law. Table S2: the fitting results of ac conductivity using Drude model. Table S3: the calculation results of real permittivity using Lorentz model. Table S4: the calculation results of equivalent circuit. Table S5: fitting results using (4). Table [file 1021368.f1.docx]

**Supplementary Materials**

**S1. Figures Section.**

**Fig. S1** shows the XRD patterns of Ag/SiO_2_ composites with different silver content. A broad diffraction peak is observed for SiO_2_ microsphere at around 21.5°, indicating the amorphous SiO_2_. It is observed that the main crystalline phase of the composites is silver, corresponding well to the standard card JCPDS No. 04-0783, which has a cubic space group of Fm-3m(225) and lattice parameters of a = b = c = 4.0862 Å (90°×90°×90°). This means that there is no any other phase peaks detected in its XRD pattern, illustrating high purity of fabricated silver by impregnation-calcination process. The crystalline peaks of silver are too high that the characteristic peaks of amorphous SiO_2_ cannot be clearly identified in the XRD patterns of composites. Besides, the EDX analysis was performed in order to distinguish the silver phase and SiO_2_ in SEM images (in **Fig. 2b** and **Fig. S1**). It indicates that the silver particles and sheets are distributed in the channels among SiO_2_ microspheres. We can see from **Fig. S1**, there are diverse morphologies of silver due to different content and agglomeration of silver nanoparticles, which is further investigated in the following discussions.

**Fig. S3** depicts the frequency dispersion of *σ*_ac_ for Ag/SiO_2_ composites. When the silver content is below *f*_c_ (i.e. SiO_2_, Ag17, Ag23 and Ag28), the *σ*_ac_ increases with increasing frequency, and shows an exponential relationship with frequency, following the power law: *σ*_ac_ = *Aω^n^*, where *ω* is the angular frequency of external electrical field, *n* (0<*n*<) is the exponent parameter. The calculation results using power law are shown as solid lines in **Fig.S3** and **Table S1**, showing good agreement with experimental data, indicating hopping conductivity behavior below percolation threshold. However, when the silver content is above *f*_c_ (i.e. Ag32, Ag35 and Ag37), the *σ*_ac_ decreases with increasing frequency, mainly attributed to skin effect of silver network. Skin effect is the characteristic behavior of conductor, described by the Drude model:

$\sigma_{ac}=\frac{\sigma_{dc}\omega_{\tau}^{2}}{\omega^{2}+\omega_{\tau}^{2}}$ (S1)

where *σ*_dc_ is the direct current (dc) conductivity, *ω*_τ_ is the damping constant and *ω*_p_ is the plasma frequency. The calculation results using **eqn (S1)** show good agreement with experimental data (solid lines in **Fig. S3** and **Table S2**), indicating a metal-like conduction behavior.

**
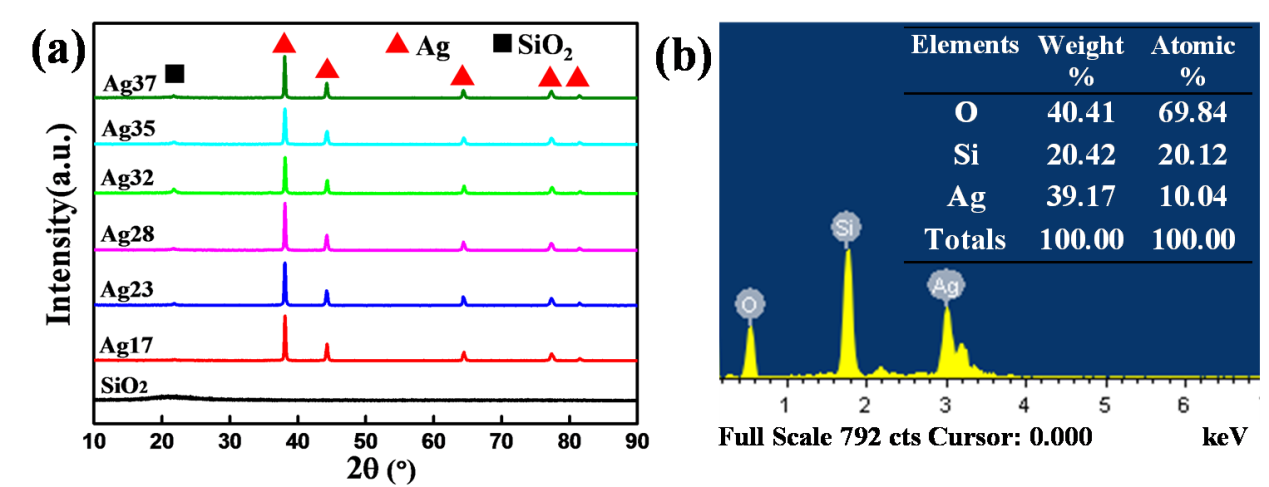
**

**Fig. S1** XRD patterns (a) of Ag/SiO_2_ composites with different silver content and EDX analysis results (b) of sample Ag23.

**Fig. S2** EDX analysis results of sample Ag23


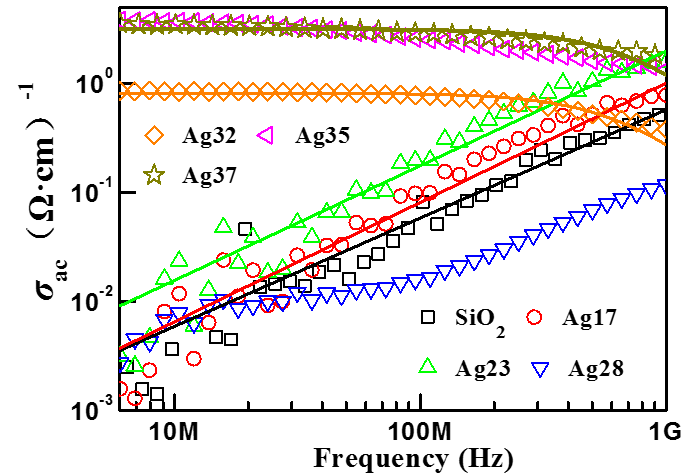


**Fig. S3** The frequency dispersion of *σ*_ac_ in 6 MHz - 1 GHz region. The solid lines are calculation results using the power law and Drude model.

The **Fig. S4** depicts the frequency dispersion of imaginary permittivity (*ε*′′) of Ag/SiO_2_ composites with different silver content. As we can see, the *ε*′′ keeps a small value for SiO_2_ bulk, Ag17 and Ag23. The *ε*′′ of Ag28 is obviously enhanced due to leakage current with increasing silver content. When silver content is above *f*_c_ (including Ag32, Ag35 and Ag37), the *ε*′′ shows a large value, and declines rapidly with increasing frequency. In fact, the dielectric loss, closely associated with frequency and filler concentration, mainly consists of the conduction loss (*ε*_c_′′) and polarization loss (*ε*_p_′′):

$\varepsilon''=\varepsilon_{p}^{' '}+\varepsilon_{c}^{' '}=\varepsilon_{p}^{' '}+\frac{\sigma_{dc}}{\omega\varepsilon_{0}}$ (S2)

Calculation operation is made using *ε*_c_′′ in **eqn (S2)**. The calculation results, especially at lower frequency region, agree well with experimental data of Ag35 and Ag32 (solid lines in **Fig. S4a**). This indicates that dielectric loss is dominant by *ε*_c_′′ at lower frequency region. However, the *ε*′′ of Ag32 starts to deviate from *ε*_c_′′ ∝ *σ*_dc_/*ω* at about 150 MHz, at 20 MHz for Ag35, and even at lower frequency for Ag37. These phenomena indicate that *ε*_p_′′ (resonance loss, relaxation loss and interfacial loss) become remarkable besides *ε*_c_′′ at high frequency region.

Dielectric loss tangent (tan*δ*) is also investigated in **Fig. S4b**. The tan*δ* shows a small value for SiO_2_ bulk, Ag17 and Ag23, but its value is obviously enhanced for Ag28 with increasing silver content. Further increasing silver content (including Ag32, Ag35 and Ag37), their tan*δ* show a relatively high value. A peak of tan*δ* is observed at 520 MHz for Ag32, while at 55 MHz for Ag35 and 10.2 MHz for Ag37. Interestingly, the frequency of the loss peaks corresponds well with the epsilon-zero points (in **Fig. 5b**). Besides, the tan*δ* of samples with negative permittivity (Ag32, Ag35 and Ag37), no longer monotonically increase with increasing silver content. For example, the *ɛ*' of Ag 32 and Ag35 are both negative below 55 MHz, the tan*δ* of Ag35 is lower than that of Ag32; the tan*δ* of Ag32 is the biggest above 83 MHz, while that of Ag37 is the smallest. Therefore, the loss of negative permittivity has prospective to be tailored and minimized by changing the content and distribution of conductive fillers in metacomposites.


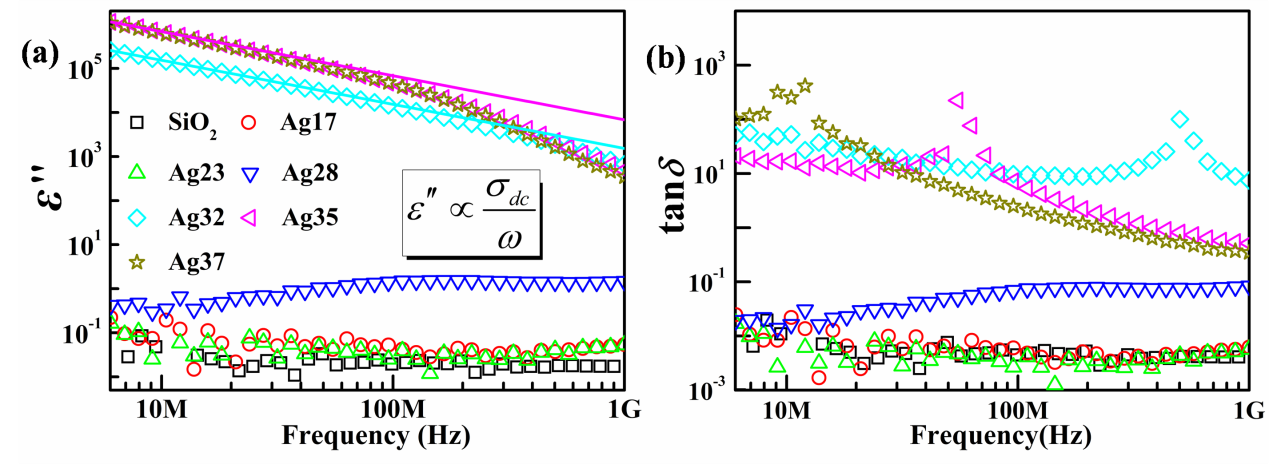


**Fig. S4** Frequency dependence of imaginary permittivity *ε*′′ (a) and dielectric loss tangent tan*δ* (b) of Ag/SiO_2_ composites with different silver content.

The frequency dispersion of phase angle *θ* is shown in **Fig. S5**. The resistance current *I*_R_ (i.e. conduction current), capacitance current *I*_C_ and inductive current *I*_L_ are expressed as:

$I_{R}=\frac{UR}{R^{2}+X^{2}}$ $I_{C}=\frac{UX_{C}}{R^{2}+X_{C}^{2}}$ $I_{L}=\frac{-UX_{L}}{R^{2}+X_{L}^{2}}$ (S3)

where *U* is the voltage of external electrical field, *R* is resistance, *X* is reactance, *X*_C_ is capacitive reactance, and *X*_L_ is the inductive reactance. The phase of *I*_R_ is synchronous with the *U*. The phase of *U* falls behind *I*_C_ by 90°, while phase of *I*_L_ falls behind *U* by 90°. Based on the phase of current, the tan*δ* and the phase angle *θ* (-90°≤*θ*≤90°) can be expressed by **eqn (S4)**:

$\tan\delta=\left|\frac{I_{R}}{I_{X}}\right|=\left|\frac{R}{X}\right|$ $\tan\theta=\frac{X}{R}$ (S4)

where *I*_X_ is the reactive current (i.e. the sum of *I*_C_ and *I*_L_). The relation between phase angle and loss angle satisfies |*δ*| + |*θ*| =90°. That is, the bigger absolute value of *θ*, the smaller dielectric loss in Ag/SiO_2_ composites. When silver content is below *f*_c_, negative *θ* is shown in **Fig. S5**, indicating capacitive character, the *I*_R_ lags behind *I*_C_ by 90°.^7^ The *θ* of SiO_2_ bulk, Ag17, Ag23 and Ag28 are near 90°, suggesting that most electric energy is stored in capacitance with low conduction loss. The low conduction loss in these four samples, according to **eqn (S2)**, further demonstrates that the dielectric loss mainly originates from polarization loss when the silver content is below *f*_c_ (in **Fig. S4**). The low conduction loss has been demonstrated by the equivalent circuit analysis, because a large parallel resistance is in their equivalent circuits (in **Fig. S6** and **Table S4**).


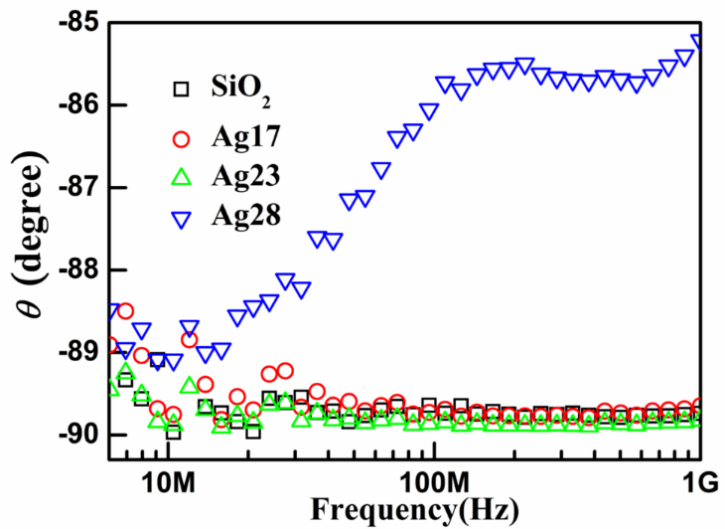


**Fig. S5** Frequency dependence of phase angle *θ* for Ag/SiO_2_ composites with different silver content at 6 MHz–1 GHz.

**Fig. S6** Nyquist plots for samples SiO_2_ bulk, Ag17 and Ag23; their results of equivalent circuit analysis. The inset is the equivalent circuit for Ag/SiO_2_ composites with different silver content. The solid lines are calculation results using equivalent circuit.

**Fig. S7** The fitting results of permeability spectra of Ag32, Ag35 and Ag37 using magnetic plasma. The red solid lines are fitting data.

**Fig. S8** The fitting results of permeability spectra of Ag32, Ag35 and Ag37 by linear fitting. The solid lines are fitting data.

**Fig. S9** The calculation results of permeability spectra of Ag17, Ag23 and Ag28 by **eqn (7)**. The solid lines are calculation data with high reliability.

The electromagnetic induction could generate magnetic loss under high-frequency electromagnetic field, and the frequency dependence of imaginary permeability (*μ*″) for Ag/SiO_2_ metacomposites is shown in **Fig. S10a**. The *μ*″ is small when the silver content is low. The *μ*″ obviously increases with increasing silver content whereas decreases with increasing frequency.


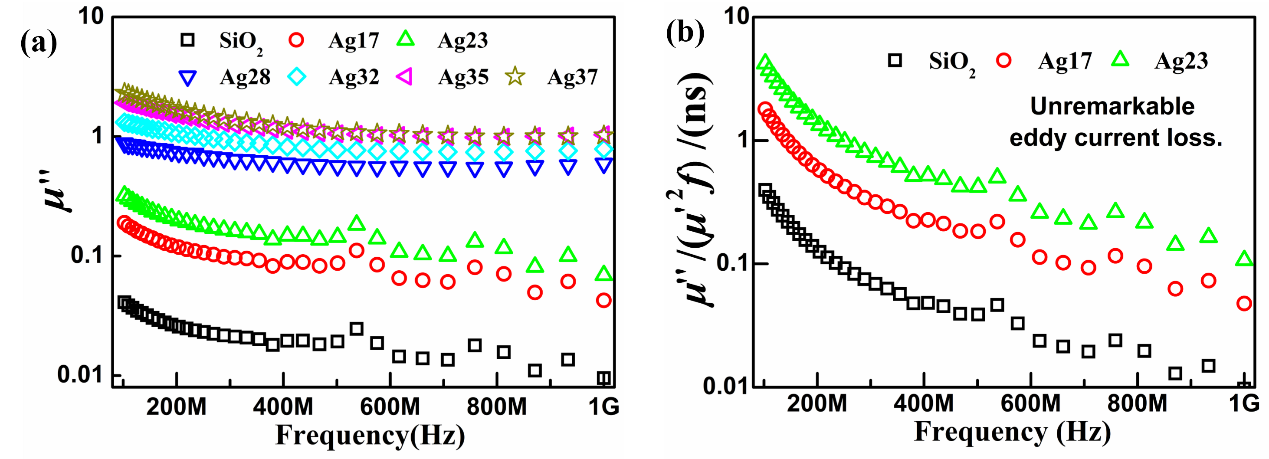


**Fig. S10** Frequency dependence of imaginary permeability (*μ*″) (a) and *μ*″/(*μ*′^2^*f*) (b) for Ag/SO_2_ composites with different silver content.

As shown in **Fig. S11**, a square slab model (200 mm × 200 mm) with different thickness *d* (*d* = 0.1, 0.25, 0.5 and 1mm) are built, perfect electric conductor (PEC) and perfect magnetic conductor (PMC) were used to simulate transverse electromagnetic wave (TEM) waveguide. The materials of square slab were set to have the experimental electromagnetic data in **Figure 5-9**. The scattering (S11 and S12) parameters were obtained, and shielding effectiveness (SE) was evaluated using **eqn S5-S9**.

$R=\left|S_{11}\right|^{2}$ (S5)

$T=\left|S_{21}\right|^{2}$ (S6)

$SE_{R}=-10\lg(1-R)$ (S7)

$SE_{A}=-10\lg\left[T/\left(1-R\right)\right]$ (S8)

$SE=SE_{R}+SE_{A}$ (S9)

**Fig. S11** The square slab model built for numerical simulation


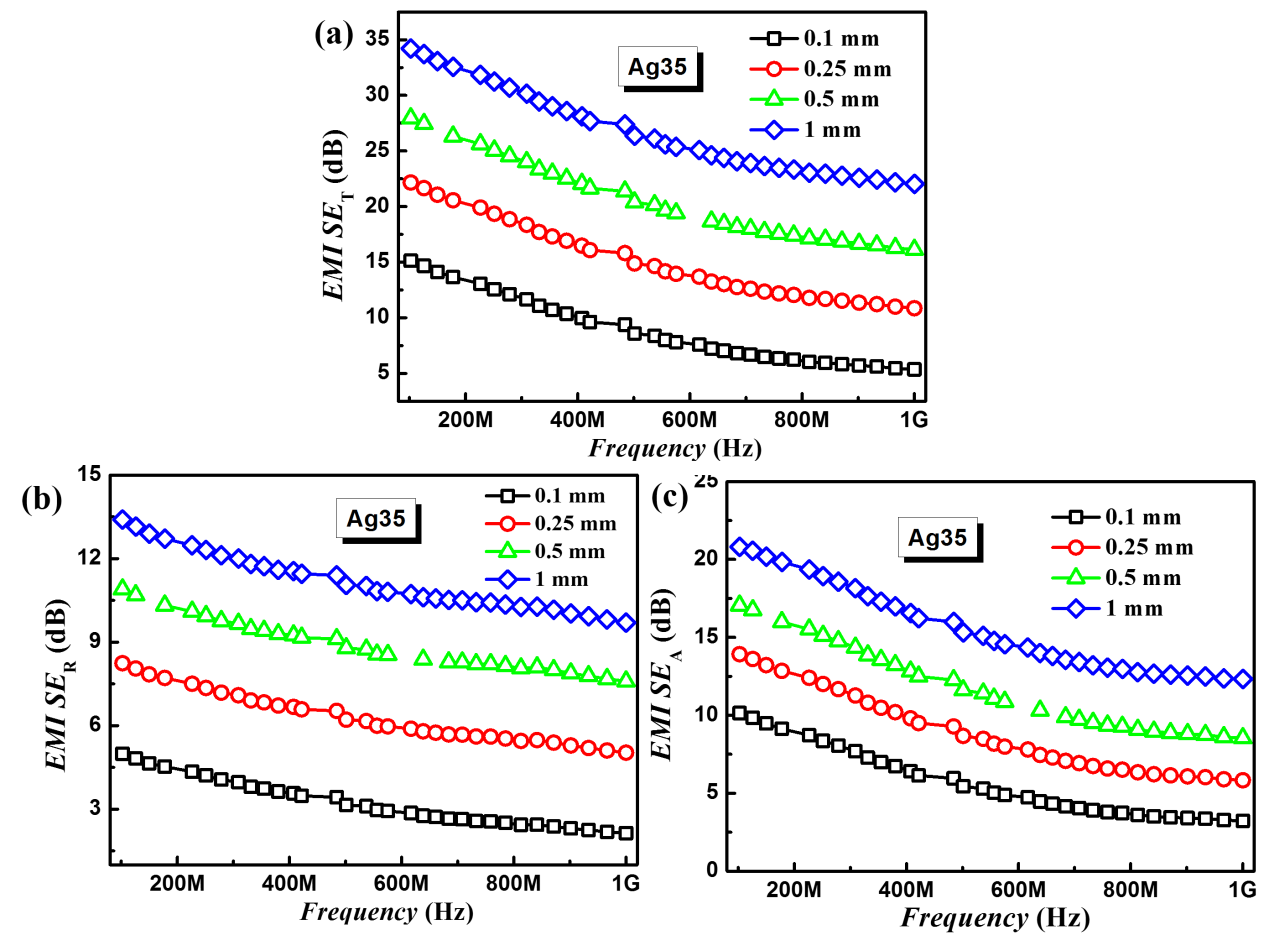


**Fig. S12** Frequency dispersion of EMI SE_T_ (a), SE_R_ (b) and SE_A_ (c) for Ag35 composites with different thickness.


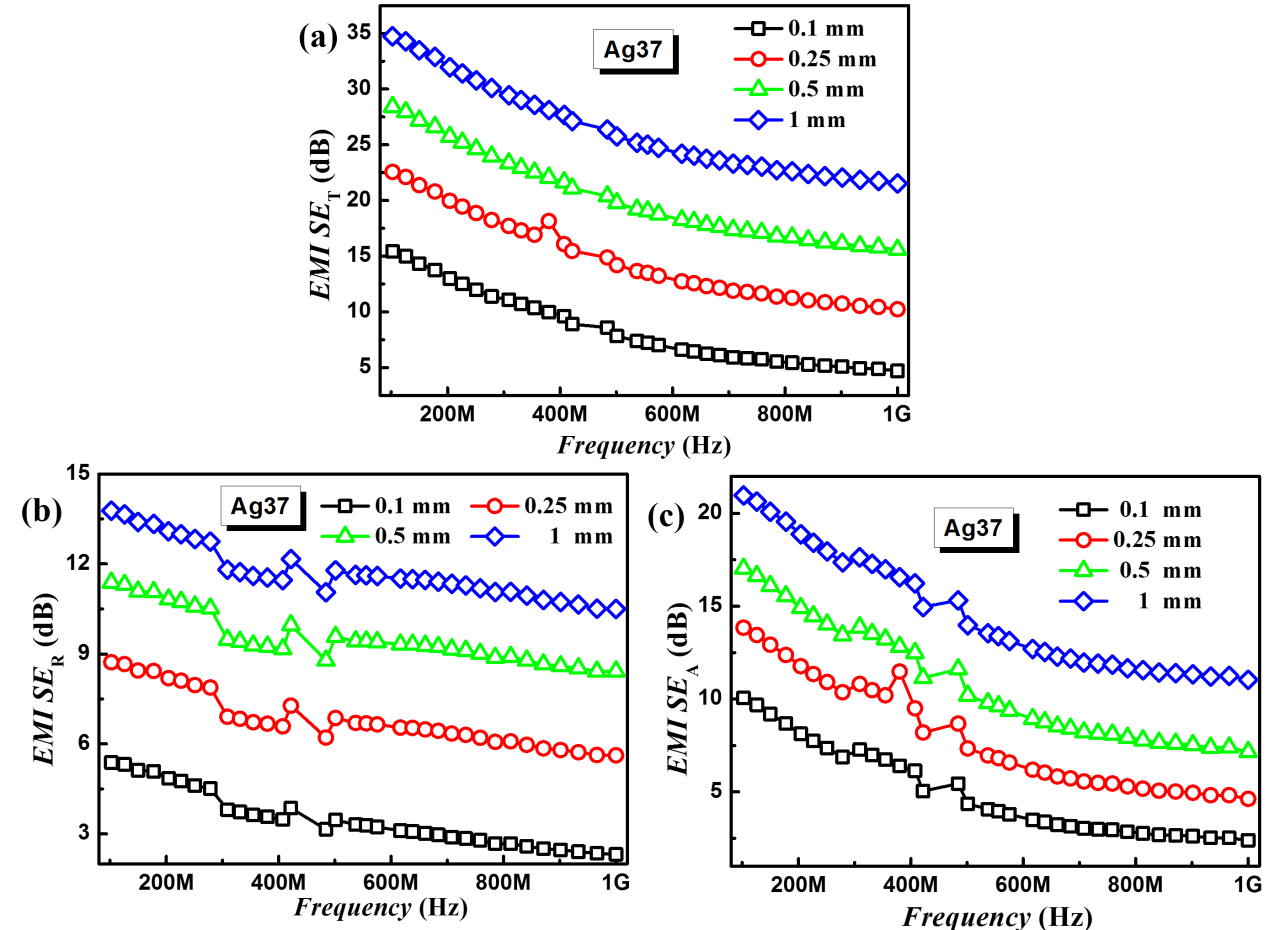


**Fig. S13** Frequency dispersion of EMI SET (a), SER (b) and SEA (c) for Ag37 composites with different thickness.

**S2. Tables Section.**

**Table S1** The fitting results of ac conductivity using power law

| Samples | Pre-exponental factor *A* | *n* | Reliability factor *R*^2^ |
| --- | --- | --- | --- |
| SiO_2_ | 9.979 × 10^-11^ | 0.99632 | 0.98219 |
| Ag17 | 1.794 × 10^-11^ | 1.09734 | 0.94074 |
| Ag23 | 9.322 × 10^-11^ | 1.0543 | 0.94974 |

**Table S2** The fitting results of ac conductivity using Drude model

| Samples | *σ*_dc_ | *ω*_τ_ | Reliability factor *R*^2^ |
| --- | --- | --- | --- |
| Ag32 | 213777 | 4.458 × 10^9^ | 0.91888 |
| Ag37 | 442397 | 4.933 × 10^9^ | 0.75074 |

**Table S3** The calculation results of real permittivity using Lorentz model

| Samples | *ω*_p_ | *Γ*_D_ | *K* | *ω*_L_ | *Γ*_L_ | *R*^2^ |
| --- | --- | --- | --- | --- | --- | --- |
| Ag35 | 6.942×10^9^ | 1.773×10^8^ | 12554.51 | 2.551×10^9^ | 1.412×10^11^ | 0.99589 |

**Table S4** The calculation results of equivalent circuit

| Samples | *R*_s_ (Ω·cm^2^) | *R*_p_ (Ω·cm^2^) | *C*_p_ (F/cm^2^) | Chi-Sq |
| --- | --- | --- | --- | --- |
| SiO_2_ | 0.3893 | 1.72 × 10^5^ | 2.343 × 10^-12^ | - 1. × 10^-4^ |
| Ag17 | 0.8027 | 3.68 × 10^5^ | 1.492 × 10^-12^ | 1.82 × 10^-5^ |
| Ag23 | 0.4015 | 7.36 × 10^5^ | 1.491 × 10^-12^ | 1.65 × 10^-5^ |

**Table S5** Fitting results using the **eqn (4)**

| **Samples** | **Fitting parameters** | | | **Reliability factor** |
| --- | --- | --- | --- | --- |
| **Ag/SiO_2_** | ***F*** | ***ω*_0_** | ***Γ*** | ***R*^2^** |
| Ag32 | 5.626×10^17^ | 9.321×10^17^ | 9.015×10^26^ | 0.81792 |
| Ag35 | 6.326×10^14^ | 1.685×10^16^ | 4.630×10^23^ | 0.73536 |
| Ag37 | 5.014×10^14^ | 1.418×10^16^ | 3.288×10^23^ | 0.73369 |

**Table S6** Fitting parameters of linear fitting

| **Samples** | **Fitting parameters** | | **Reliability factor** |
| --- | --- | --- | --- |
| **CAs** | **Intercept** | **Slope** | ***R*^2^** |
| Ag32 | 0.73713 | -4.77×10^-10^ | 0.97542 |
| Ag35 | 0.46714 | -4.75×10^-10^ | 0.97451 |
| Ag37 | 0.40408 | -5.26×10^-10^ | 0.97462 |

**Table S7** The calculation results of **eqn (22)**

| Samples | *a* | *b* | Reliability factor *R*^2^ |
| --- | --- | --- | --- |
| Ag17 | 1.05569 | -3.578 × 10^-6^ | 0.99745 |
| Ag23 | 0.89451 | -2.913 × 10^-6^ | 0.99772 |
| Ag28 | 0.84936 | -6.563 × 10^-6^ | 0.99768 |
| Ag32 | 0.91899 | -1.963 × 10^-5^ | 0.99745 |
| Ag35 | 0.64809 | -1.962 × 10^-5^ | 0.99724 |
| Ag37 | 0.60675 | -2.165 × 10^-5^ | 0.99731 |
